# Correlating dermatoscopic features with immunohistochemical markers in basal cell carcinoma: a comprehensive analysis of 100 cases in Caucasian population

**DOI:** 10.3389/fonc.2024.1429865

**Published:** 2024-09-16

**Authors:** Jacek Calik, Natalia Sauer, Piotr Giedziun, Aleksandra Piotrowska, Maciej Tumiłowicz, Andrzej Wojnar, Piotr Dzięgiel

**Affiliations:** ^1^ Department of Clinical Oncology, Wroclaw Medical University, Wrocław, Poland; ^2^ Old Town Clinic, Wroclaw, Poland; ^3^ Faculty of Pharmacy, Wroclaw Medical University, Wroclaw, Poland; ^4^ Faculty of Information and Communication Technology, Wrocław University of Science and Technology Centre for Scientific and Technical Knowledge and Information, Wrocław, Poland; ^5^ Division of Histology and Embryology, Department of Human Morphology and Embryology, Wroclaw Medical University, Wroclaw, Poland; ^6^ Department of Haematology, Blood Neoplasms and Bone Marrow Transplantation, Medical University of Wrocław, Wroclaw, Poland; ^7^ Department of Preclinical Sciences, Pharmacology and Medical Diagnostics, Wrocław University of Science and Technology, Wrocław, Poland; ^8^ Department of Human Biology, Faculty of Physiotherapy, Wroclaw University of Health and Sport Sciences, Wroclaw, Poland

**Keywords:** basal cell carcimoma (BCC), dermatoscopy, CD31, CD34, Melan-A, D2-40

## Abstract

**Background:**

Basal Cell Carcinoma (BCC) is the most common form of skin cancer, characterized by its low metastatic potential yet considerable diversity in clinical and dermatoscopic presentation. Advances in dermatoscopy have significantly improved the early detection of BCC, revealing specific patterns that guide diagnosis and management. Parallelly, immunohistochemical markers have been explored for their potential to elucidate the underlying tumor biology and prognosis, with particular focus on angiogenesis, melanocytic activity, and lymphangiogenesis.

**Objective:**

This study aims to investigate the correlations between dermatoscopic features and the immunohistochemical expressions of CD34, CD31, Melan-A, and D2-40 in BCC, through a comprehensive analysis of 100 cases We sought to determine whether visual dermatoscopic patterns correlate with the molecular characteristics defined by immunohistochemical staining, potentially enhancing diagnostic accuracy.

**Methods:**

A total of 100 cases of clinically and histopathologically confirmed BCC were prospectively analyzed, employing standard dermatoscopic techniques for lesion evaluation and immunohistochemical staining for CD34, CD31, Melan-A, and D2-40 to assess tumor angiogenic potential, melanocytic activity, and lymphangiogenesis. The study was conducted with adherence to ethical standards and informed consent from all participants.

**Results:**

Dermatoscopic examination revealed a variety of vascular patterns and pigmented features across different BCC anatomical locations. However, the comprehensive correlation analysis predominantly found a lack of significant associations between dermatoscopic appearances and expressions of the targeted immunohistochemical markers, with the notable exception of a correlation between observed hemorrhage and the Melan-A marker.

**Conclusions:**

The lack of significant correlations between dermatoscopic features and immunohistochemical marker expressions in BCC suggests that the biological behavior and angiogenic, melanocytic, and lymphangiogenic activities within BCC lesions may be influenced by factors beyond those assessed in this study. Despite the exploratory nature of these findings, they underscore the complexity of BCC biology and highlight the need for further research incorporating additional markers and advanced imaging techniques.

## Introduction

Basal Cell Carcinoma (BCC) is the subtype of keratinocyte carcinoma, notorious for its high prevalence but low metastatic potential (1, 2). BCC is initiated within the basal layer of the epidermis, particularly arising from cells located in the interfollicular epidermis, underscoring its origin from the non-follicular regions of the epidermal basal layer (3). While the majority of BCCs are categorized as “routine” or low-risk and are straightforward to manage, approximately between 0.0028 and 0.55 of all BCC cases exhibit aggressive local behavior and metastasis (4). Dermatoscopic techniques have revolutionized the early detection of BCC, offering a non-invasive window into the microarchitecture of cutaneous tumors (5–7).

BCC exhibits specific dermatoscopic patterns crucial for its identification, encompassing vascular features such as arborizing vessels and fine telangiectasia, which signify the neoplasm’s angiogenic activity. Morphological diversity is highlighted by pigmentation markers including blue-gray ovoid nests and globules, indicative of melanin aggregation within tumor nests. Dermatoscopic indicators such as maple leaf-like areas and spoke wheel structures suggest complex growth patterns, while ulceration and erosions reflect the tumor’s invasive capacity and surface disruption (6, 8).

BCC manifests in various histopathological subtypes, including nodular and superficial forms, each characterized by distinct morphological features and patterns of growth (1).

Recent investigations have deepened our understanding of the intricate relationship between dermatoscopic patterns and the histopathological landscape of BCC (7). The significance of immunohistochemical markers such as CD31, CD34, Melan-A, and D2-40, provide unique insights into the tumor’s biological behavior and prognosis.

CD31 and CD34 are endothelial markers used to assess microvascular density (MVD) within tumors, offering insights into BCC’s angiogenic potential (7). Elevated MVD, indicated by CD31 and CD34 expression, correlates with aggressive tumor behavior, suggesting a potential mechanism for BCC growth and metastasis potential. These markers have been instrumental in distinguishing between BCC subtypes, with higher MVD observed in more invasive forms. In the study by Park et al., immunohistochemical analysis was conducted using Melan-A and HMB-45 to identify melanocytes in samples of BCC (9). Melan-A, along with HMB-45, was used for staining melanocytes and melanophages, aiming to identify and quantify the presence of melanin and melanocytic cells in the tumor tissue. The results indicated an inverse correlation between pigmentation and the aggressiveness of BCC, suggesting that higher values of dermoscopic pigmentation, melanin, and areas stained by Melan-A are associated with a lower likelihood of tumor infiltration into the middle and lower layers of the tissue. D2-40 is predominantly employed to delineate lymphatic structures, including the identification of lymphovascular invasion, and to ascertain lymphatic differentiation within vascular neoplasms; it exhibits positivity in primary cutaneous adnexal neoplasms (10).

The correlation between dermoscopy and histopathology represents a new pathway in the diagnosis and management of skin cancers (11). dermoscopy significantly enhances the sensitivity and specificity for detecting skin cancers, enabling the identification of smaller and thinner malignancies and allowing for more precise selection of lesions for excision. This technique aids clinicians in distinguishing between benign and malignant lesions through specific dermoscopic structures that often have direct histopathologic counterparts, facilitating the prediction of histologic findings and informing management and treatment choices for various skin cancers. Several studies have shown that specific dermoscopic features correspond to distinct histopathological alterations in skin cancers (12). For instance, these include melanin within the tumor mass and surrounding dermis, reflecting the localization of pigment accumulation that is crucial for the accurate diagnosis of pigmented BCCs (13). The detailed correlation between dermoscopic observations and their histopathologic counterparts facilitates a better understanding of the disease process (14, 15). It not only aids in distinguishing melanoma and BCC from benign lesions but also assists in determining the subtype of skin cancer, which can influence management decisions. For instance, the presence of ulceration, specific colors, and vascular patterns in dermoscopy can direct the clinician towards a more precise histopathological examination, ensuring that diagnostic biopsies are representative of the lesion’s most diagnostically relevant areas.

The aim of this work is to investigate the correlations between dermatoscopic features and immunohistochemical expressions of CD34, CD31, Melan A, and D2-40 in BCC through a comprehensive analysis of 100 cases, aiming to elucidate the relationship between the visual dermatoscopic patterns observable in non-invasive examinations and the underlying molecular and cellular characteristics defined by immunohistochemical staining.

## Materials and methods

### Study population

The study involved 100 Basal Cell Carcinoma (BCC) cases diagnosed at the Old Town Clinic between December 2022 and May 2023. The inclusion criteria specified that participants must have a clinical and histopathological diagnosis of BCC and be at least 18 years old. The study was conducted prospectively. Informed consent was obtained from all participants, and the study adhered to the ethical principles outlined in the Helsinki Declaration II.

### Dermatoscopic evaluation

We performed a clinical evaluation of each skin lesion using the Fotofinder Medicam 1000 and 800 devices and identified lesion dermatoscopic patterns and types of vessels. The dermatoscopic terminology utilized in this study aligns with the standard dermatoscopic terminology established by a consensus of experts on behalf of the International Dermoscopy Society (16). To assess vascular morphology, minimal pressure was applied.

### Immunohistochemical evaluation

Immunohistochemical reactions were performed on 4-µm thick paraffin sections using Autostainer Link48 (Agilent, Santa Clara, CA, USA). To deparaffinize, rehydrate and unmask the epitopes the sections were boiled in Target Retrieval Solution, high pH (pH 9, 97°C, 20 min) (Agilent) using PT Link Platform (Agilent) and, subsequently, cooled in EnVision FLEX Wash Buffer (Tris-buffered saline solution containing Tween 20). In order to block endogenous peroxidase activity slides were incubated with EnVision FLEX Peroxidase- Blocking Reagent (5 min; RT, Agilent). Then, the sections were washed in EnVision FLEX Wash Buffer. Monoclonal mouse anti-human antibodies against CD31 Endothelial Cell (RTU, clone JC70A, IR610, Agilent), CD34 Class II (RTU, clone QBEnd10, IR632, Agilent), Podoplanin (RTU, clone D2-40, IR606, Agilent) and Melan-A (RTU, clone A103, IR633, Agilent) were applied for 20 min at RT. After washing in EnVision FLEX Wash Buffer, the sections were incubated with EnVision FLEX/HRP secondary antibodies (20 min, RT). In the next step, sections were washed in EnVision FLEX Wash Buffer and the substrate for peroxidase – diaminobenzidine (DAB) was applied for 10 min at RT. Subsequently, all the slides were counterstained with FLEX Hematoxylin (Agilent) for 5 min at RT, dehydrated in ethanol alcohol (70%, 96%, 99,8%) and xylene. Finally, the preparations were mounted in SUB-X Mounting Medium (Agilent).

The immunohistochemical reactions were evaluated using a BX-41 light microscope (Olympus, Tokyo, Japan) by two independent researchers. CD31, CD34 and podoplanin (D2-40) expression was observed in cytoplasm of endothelial cells. Initially, the sections were examined under low magnification (x100) to identify three areas with the highest vascular density (hot-spots). Subsequently, under x400 magnification stained vessels were counted. An average score was estimated for three hot-spots.

Melan-A expression was evaluated using a semi-quantitative five-grade scale based on the percentage of positively stained cancer cells showing a brown reaction in the cytoplasm: 0 – no reaction, 1 point – 1-10% cells stained; 2 points – 11-25%; 3 points – 26-50%; and 4 points – 51-100%.

### Statistical analyses

All statistical analyses were conducted using the R language (version 4.1) and Python (version 3.10). Means, standard deviations, medians, and interquartile ranges were calculated for continuous variables such as age. Frequencies and percentages were computed for categorical variables such as gender and anatomical location of lesions. The Spearman’s rank correlation coefficient was used to assess the relationships between dermatoscopic features and the expressions of immunohistochemical markers (CD34, CD31, Melan-A, and D2-40) due to the non-parametric nature of the data. The Mann-Whitney U test was employed to compare the distribution of dermatoscopic features between groups defined by the expression levels of the immunohistochemical markers. The Mann-Whitney U test was used for comparing non-normally distributed data. The Shapiro-Wilk test was performed to assess the normality of continuous variables. The t-test was applied for normally distributed variables to compare means between two groups. The Levene’s test was conducted to check the equality of variances before performing t-tests. Scatter plots were generated to visualize the correlation between age and the expression levels of specific biological markers. Comparative analysis tables and visual representations of dermoscopic features were created to enhance the interpretation of the findings. The statistical significance was set at p < 0.05 for all tests. All analyses and visualizations were conducted using the respective libraries in R and Python to ensure robust and reproducible results.

### Study assumptions

We aimed to investigate the correlations between dermatoscopic features and the expressions of CD34, CD31, Melan A, and D2-40 (Podoplanin), briefly detailing their respective roles. CD34 is known for its role in marking the presence of endothelial progenitor cells, important for the formation of new blood vessels (17). CD31, also known as PECAM-1, is involved in the endothelial cell-cell adhesion, playing a critical role in vascular integrity and the inflammatory response (18). Melan-A is a marker of melanosomal maturation, therefore it reveals primarily melanin (19). D2-40 (Podoplanin) is recognized for its expression in lymphatic endothelial cells, implicated in the processes of lymphangiogenesis and tumor metastasis. We anticipated potential correlations between CD31 and CD34 with the presence of blood vessels. Additionally, we expected correlations such as Melan A with pigmentary structures like gray-blue nodules, and D2-40 (Podoplanin) with white structures, including white circles, scales, featureless white areas, rosettes, white nodules, yellow-white nodules, and white lines. Structures were correlated with their location and with IHC findings.

## Results

### Dermoscopic features

In this study, male participants accounted for 60% of the total population, whereas females comprised 40%. Participant ages ranged from a minimum of 33 to a maximum of 93 years, with the group’s average age being approximately 67.57 years and a standard deviation of 15.20 years. Among female participants, ages spanned from 38 to 93 years, averaging around 61.63 years with a standard deviation of 15.02 years. Conversely, the age range for male participants was between 33 and 88 years, with an average age of about 71.53 years and a standard deviation of 14.09 years. All patients were of Caucasian ethnicity. We evaluated the dermatoscopic features of BCC lesions across different anatomical locations: 53% were located on the face, 35% on the trunk, and 12% on the limbs. Dermatoscopic examination revealed a variety of vascular patterns among these lesions (**Table 1**). In the study cohort, nodular basal cell carcinoma (BCC) constituted approximately 21% of the cases, while the superficial subtype accounted for the remaining 79%. Notably, the histopathological evaluation did not include an assessment of infiltration depth. However, clinical examinations supported the classification into these specific subtypes.

**Table 1:** Distribution and Vascular Characteristics of Basal Cell Carcinoma (BCC) by Anatomical Location.

Vascular Characteristics	BCC in total	BCC of facial skin	BCC of trunk skin	BCC of limb skin
Long linear vessels	63 (63.00%)	32 (61,54%)	23 (65.71%)	8 (66.67%)
Short linear vessels	51 (51.00%)	26 (50.00%)	20 (57.14%)	5 (41.67%)
Branched linear vessels	78 (78.00%)	43 (82.6%)	27 (77.14%)	8 (66.67%)
Loop vessels	31 (31.00%)	14 (27.45%)	11 (31.43%)	6 (50.00%)
Curved linear vessels	9 (9.00%)	8 (15.69%)	0 (0.00%)	1 (8.33%)
Serpentine linear vessels	13 (13.00%)	8 (15.38%)	5 (14.29%)	0 (0.00%)
Helical linear vessels	0 (0.00%)	0 (0.00%)	0 (0.00%)	0 (0.00%)
Glomerular vessels	18 (18.00%)	3 (5.88%)	14 (40.00%)	1 (8.33%)
Dot vessels	7 (7.00%)	0 (0.00%)	5 (14.29%)	2 (16.67%)
Clod vessels	3 (3.00%0	2 (3.92%)	0 (0.00%)	1 (8.33%)
Pink structureless areas	63 (63.00%)	33 (63.46%)	23 (65.71%)	7 (58.33%)

**Figure 1:**
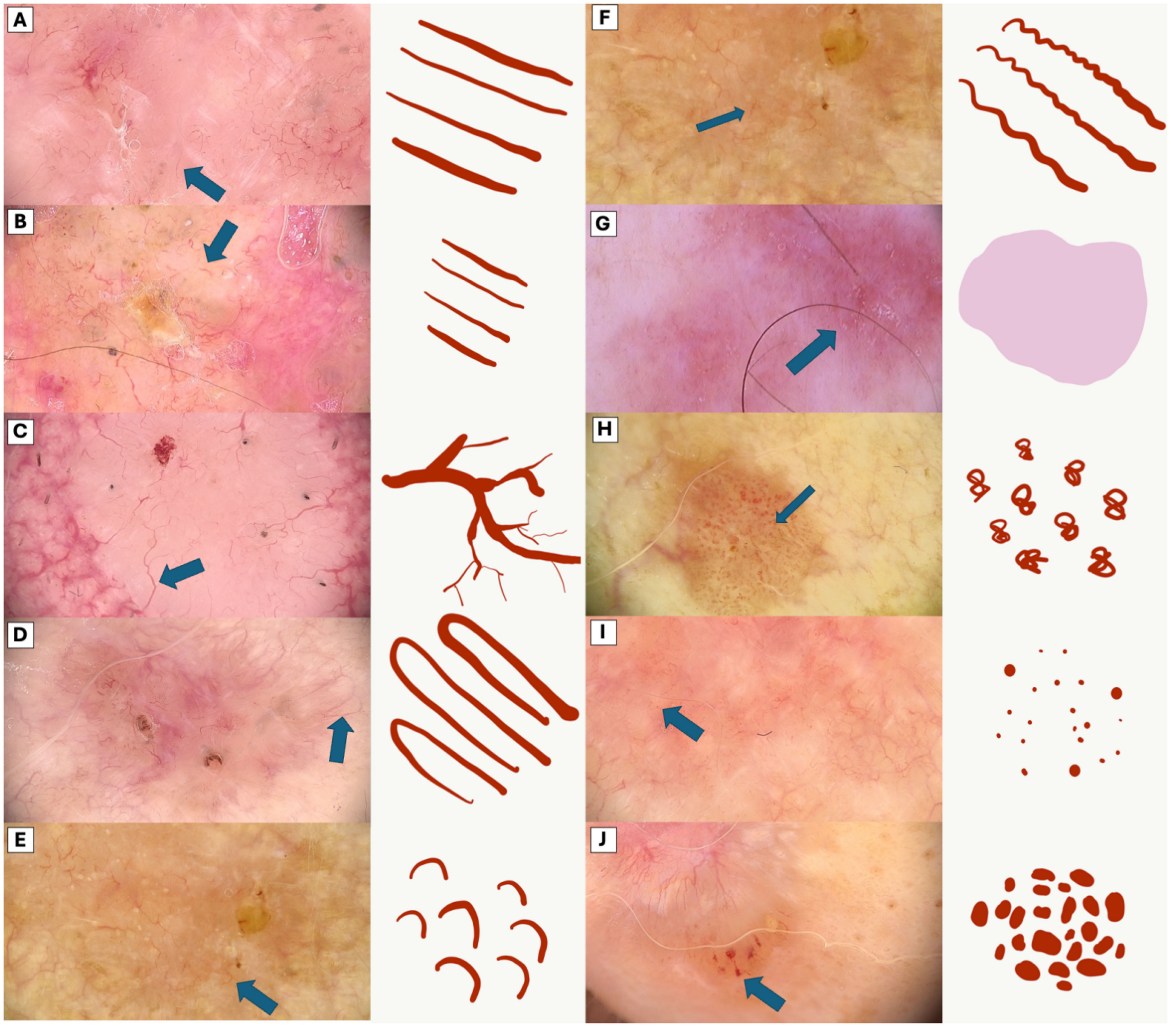
Comparative Dermoscopic of Vascular Patterns. **(A)** Long linear vessels **(B)** Short linear vessels, **(C)** Branched linear vessels, **(D)** Loop vessels, **(E)** Curved linear vessels, **(F)** Serpentine linear vessels, **(G)** Pink structureless areas, **(H)** Glomerular vessels, **(I)** Dot vessels, **(J)** Clod vessels.

To provide a visual representation and enhance understanding of the subject, **Figure 1** illustrates the diverse appearances and characteristics associated with the vascular patterns.

In the comprehensive analysis of BCC by anatomical location, both pigmented and white dermoscopic features are meticulously outlined in **Table 2**.

**Table 2:** Distribution and Dermoscopic Features of Basal Cell Carcinoma (BCC) by Anatomical Location.

Dermoscopic Features	BCC in total	BCC of facial skin	BCC of trunk skin	BCC of limb skin
Segmentally arranged pigment lines	6 (6.00%)	1 (1.96%)	2 (5.71%)	3 (25.00%)
Radially arranged pigment lines	2 (2.00%)	1 (1.96%)	0 (0.00%)	1 (8.33%)
Radially arranged pigment lines	0 (0.00%)	0 (0.00%)	0 (0.00%)	0 (0.00%)
Small blue-gray globules	34 (34.00%)	13 (25.49%)	16 (45.71%)	5 (41.67%)
Large blue-gray globules	6 (6.00%)	0 (0.00%)	3 (8.57%)	3 (25.00%)
Brown dots	17 (17.00%)	7 (13.73%)	8 (22.86%)	2 (16.67%)
Hyperpigmented microcircles	3 (3.00%)	2 (3.92%)	0 (0.00%)	1 (8.33%)
White lines	43 (43.00%)	22 (43.14%)	15 (42.86%)	6 (50.00%)
Follicular openings	6 (6.00%)	6 (11.76%)	0 (0.00%)	0 (0.00%)
Four bright white points	10 (10.00%)	9 (17.65%)	0 (0.00%)	1 (8.33%)
Small white structureless areas	47 (47.00%)	24 (47.06%)	18 (51.43%)	5 (41.67%)
Large white structureless areas	3 (3.00%)	2 (3.92%)	1 (2.86%)	0 (0.00%)
White-yellow globules	9 (9.00%)	8 (15.69%)	1 (2.86%)	0 (0.00%)
Ulceration (orange clod)	33 (33.00%)	16 (30.77%)	15 (42.86%)	2 (16.67%)
Scale	13 (13.00%)	12 (23.08%)	1 (2.86%)	0 (0.00%)
Milia-like cysts	7 (7.00%)	5 (9.80%)	0 (0.00%)	2 (16.67%)
Hemorrhage	14 (14.00%)	11 (21.57%)	3 8.57%	0 (0.00%)

**Figure 2:**
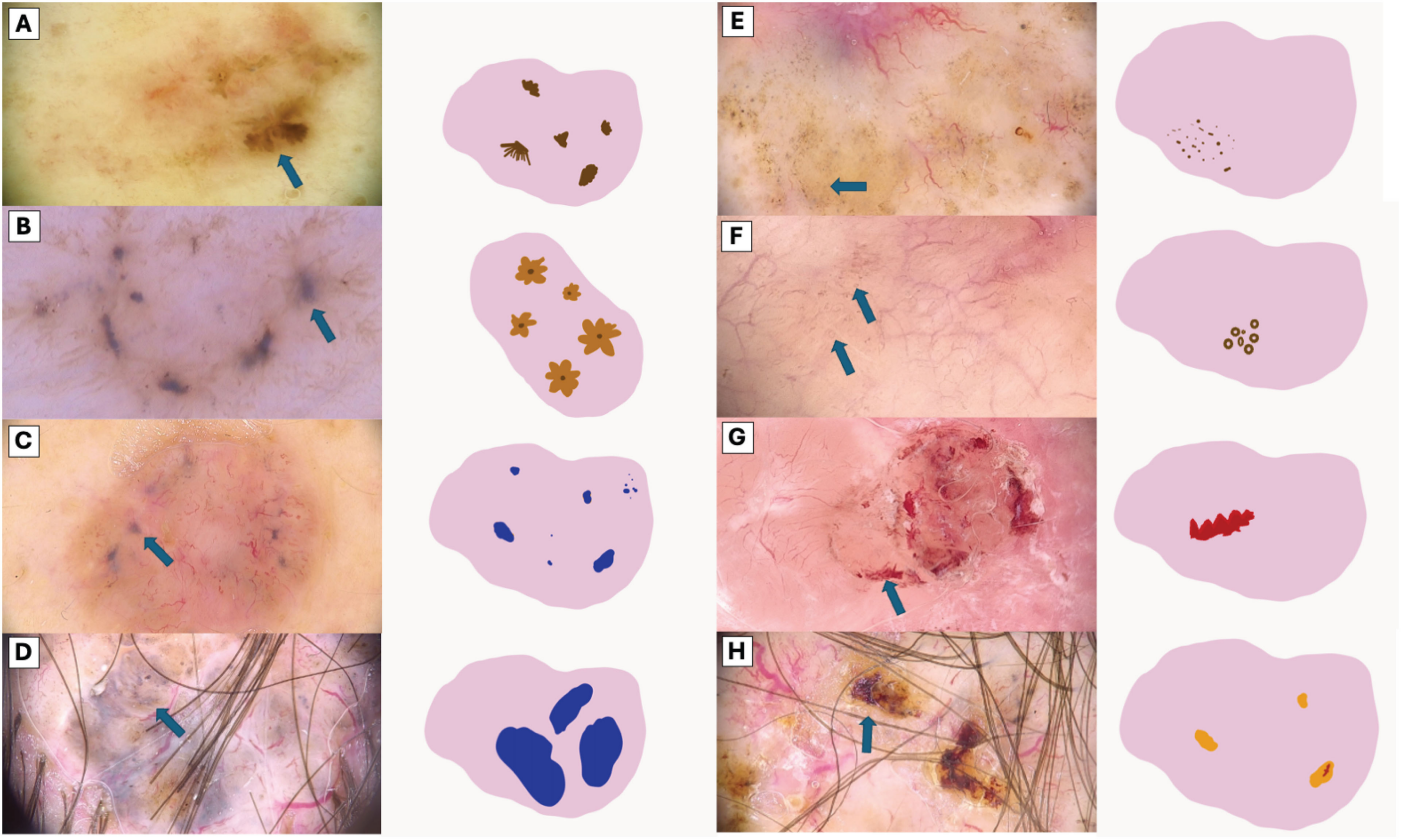
Visual Representation of Dermoscopic Structures. **(A)** Segmentally arranged pigment lines, **(B)** Radially arranged pigment lines, **(C)** Small blue-gray globules, **(D)** Large blue-gray globules, **(E)** Brown dots, **(F)** Brown microcircles **(G)** Hemorrhage, **(H)** Ulceration (orange clod).

**Figure 3:**
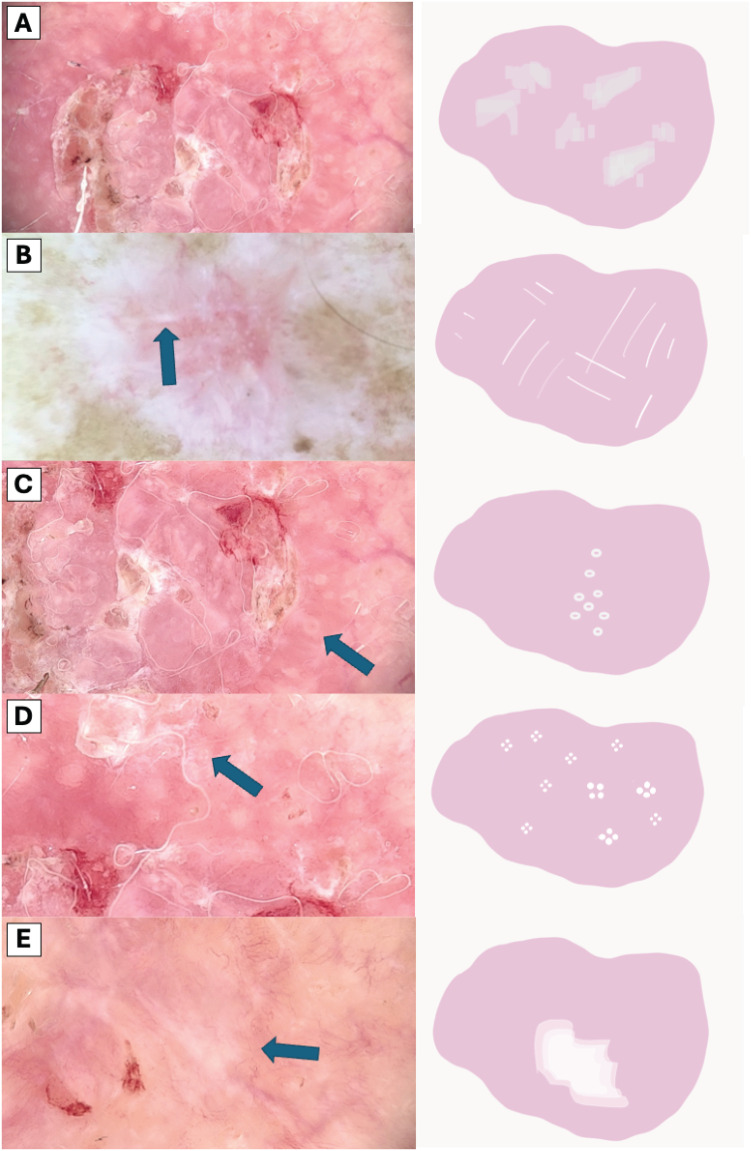
Comparative Dermoscopic of White Structures. **(A)** Scale, **(B)** White lines, **(C)** Hyperpigmented microcircles, **(D)** Four bright white points, **(E)** Large white structureless areas.

The visual representation of dermatoscopic features are illustrated in **Figures 2** and **3**.

### Clinicopathological features

**Figure 4:**
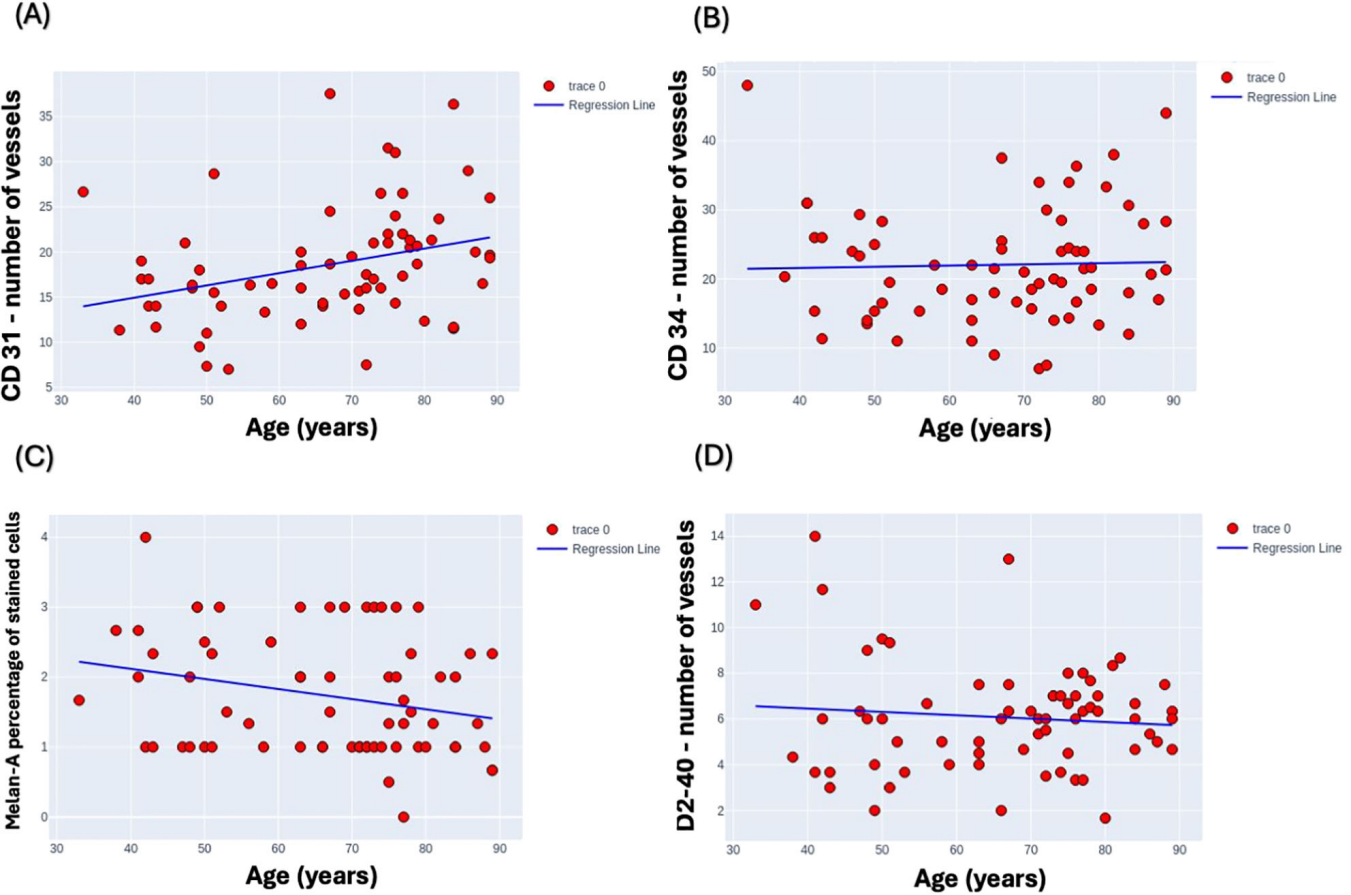
Scatter plots depicting the variability of marker expression levels by age. **(A)** CD31, **(B)** CD34, **(C)** Melan-A, and **(D)** D2-40 marker levels are plotted against age range from 30 to 90 years to analyze potential correlations. Melan-A was assessed by percentage of stained cells, CD31, CD34, D2-40 were assessed by number of vessels in the field of view under x400. Each plot demonstrates a distinct dispersion of data points, indicating the diverse expression profiles across the studied age spectrum.

These scatter plots were generated to analyze potential correlations between age and the expression levels of specific biological markers, examining the variability of markers CD31, CD34, Mela A, and D240 across a wide age range (**Figure 4**). Upon analysis, it became evident that there were no discernible trends or patterns in the data, indicating a lack of significant correlation between the age of the subjects and the expression levels of these specific markers. This suggests that, within the scope of our study, age does not influence the variability of these markers in a statistically significant manner. Specifically, there was no significant correlation between D2-40 mean levels and age, with an R-squared value of 0.009, and similarly, no significant correlation between CD34 mean levels and age, with an R-squared value of 0.001. In contrast, a moderate, statistically significant positive correlation was observed between CD31 mean levels and age, with an R-squared value of 0.104, and a weak but statistically significant negative correlation was observed between Melan-A mean levels and age, with an R-squared value of 0.065.

We aimed to elucidate the relationship between dermatoscopic patterns and immunohistochemical markers. Our findings, as detailed in the correlation **Table 3**, reveal that most correlations between dermatoscopic features and immunohistochemical markers were not statistically significant. However, a noteworthy exception emerged in the correlation between hemorrhage observed in dermoscopy and the Melan-A marker, which demonstrated a statistically significant correlation with a U-value of 152.5000 and a p-value of 0.0418.

**Table 3:** Dermoscopy correlation with histopathology.

	Dermoscopic feature	Melan-A – percentage of stained cells	D2-40 – number of vessels in the field of view under x400	CD34 – number of vessels in the field of view under x400	CD31 – number of vessels in the field of view under x400
0	Long linear vessels	U=594.5000, p-value=0.3592**	U=562.0000, p-value=0.6353**	t-value=-0.6721, p-value=0.5039*	U=541.5000, p-value=0.8356**
1	Short linear vessels	U=523.5000, p-value=0.6432**	U=482.0000, p-value=0.3296**	t-value=0.3694, p-value=0.7130*	t-value=-0.3956, p-value=0.6937*
2	Branched linear vessels	U=334.5000, p-value=0.3965**	U=431.0000, p-value=0.5415**	t-value=-0.1568, p-value=0.8759*	t-value=0.4299, p-value=0.6687*
3	Loop vessels	U=480.0000, p-value=0.8427**	U=453.5000, p-value=0.5833**	t-value=0.0132, p-value=0.9895*	t-value=0.3183, p-value=0.7513*
4	Curved linear vessels	U=123.5000, p-value=0.4484**	U=169.0000, p-value=0.7468**	U=173.5000, p-value=0.7156**	U=162.0000, p-value=0.8767**
5	Serpentine linear vessels	U=334.0000, p-value=0.6582**	U=245.0000, p-value=0.2891**	t-value=0.6091, p-value=0.5446*	t-value=0.4639, p-value=0.6443*
6	Glomerular vessels	U=280.5000, p-value=0.3832**	U=230.0000, p-value=0.9151**	t-value=0.7459, p-value=0.4584*	U=259.5000, p-value=0.6564**
7	Dot vessels	U=105.0000, p-value=0.5782**	U=91.5000, p-value=0.3672**	t-value=-0.4113, p-value=0.6822*	U=86.0000, p-value=0.2957**
8	Small blue-gray globules	U=533.5000, p-value=0.7148**	U=411.5000, p-value=0.2135**	t-value=-1.2065, p-value=0.2319*	U=377.0000, p-value=0.0896**
9	Brown dots	U=310.0000, p-value=0.6586**	U=241.0000, p-value=0.4430**	t-value=-1.5407, p-value=0.1282*	U=236.0000, p-value=0.3933**
10	White lines	U=505.0000, p-value=0.6513**	U=543.0000, p-value=0.9745**	t-value=0.6634, p-value=0.5094*	U=593.0000, p-value=0.5020**
11	Four bright white points	U=208.5000, p-value=0.3271**	U=284.5000, p-value=0.6717**	t-value=0.1447, p-value=0.8854*	U=304.5000, p-value=0.4290**
12	Small white structureless areas	U=484.5000, p-value=0.3468**	U=573.0000, p-value=0.8550**	U=581.0000, p-value=0.9313**	U=708.5000, p-value=0.0592**
13	Pink structureless areas	U=634.0000, p-value=0.1153**	U=483.0000, p-value=0.6702**	t-value=1.0703, p-value=0.2884*	U=550.0000, p-value=0.6612**
14	White-yellow globules	U=264.0000, p-value=0.2610**	U=218.0000, p-value=0.8776**	t-value=-0.9374, p-value=0.3520*	U=184.5000, p-value=0.6082**
15	Ulceration (orange clod)	U=532.5000, p-value=0.3839**	U=592.0000, p-value=0.0952**	U=554.0000, p-value=0.4256**	t-value=1.2086, p-value=0.2312*
16	Scale	U=253.0000, p-value=0.8876**	U=183.5000, p-value=0.1559**	t-value=0.6889, p-value=0.4933*	U=252.0000, p-value=0.8758**
17	Milia-like cysts	U=175.0000, p-value=0.6335**	U=174.5000, p-value=0.6496**	U=118.0000, p-value=0.3593**	U=120.5000, p-value=0.4171**
18	Hemorrhage	U=152.5000, p-value=0.0418**	U=268.5000, p-value=0.8974**	t-value=0.9031, p-value=0.3698*	U=265.5000, p-value=0.9413**

*T-test (normality checked with Shapiro-Wilk test, quality of variances checked with Levene’s test).

**Mann-Whitney U Test.

## Discussion

The integration of dermatoscopy into the clinical evaluation of skin lesions represents a significant advancement in diagnostic accuracy, providing a non-invasive glimpse into the morphological subtleties that may indicate whether a lesion is benign or malignant (20–22). Correlating histology with dermoscopy provides several benefits (23). Firstly, it enhances diagnostic accuracy by evaluating lesions comprehensively, aiding in selecting optimal biopsy sites, and guiding pathologists toward the most representative areas. Secondly, it offers prognostic insights, such as identifying mitotic activity, Breslow thickness, dermal invasion, or lymph node metastases, which are crucial for treatment planning and patient management in various skin conditions, including melanocytic neoplasms. Additionally, dermoscopy assists in predicting histologic subtypes of skin cancers, guiding appropriate treatment strategies, and even anticipating treatment response, thereby optimizing therapeutic outcomes. Furthermore, dermoscopic observations are meaningful to histopathological findings, enabling a more precise diagnosis of BCC subtypes and a deeper comprehension of dermoscopic patterns that are atypical for the disease (24).

Recent research highlights the significant role of correlations between immunohistochemical markers and dermoscopic features in diagnostic improvement (23). In lung cancer studies, the expression of CD31, CD34, and CD105 was found to be closely related to the type of tissue, differing significantly between cancerous tissues, pre-cancerous tissues, and healthy tissues adjacent to tumors, indicating their potential as indicators of tumor malignancy (23, 25). For cutaneous lymphangioma circumscriptum, studies combining dermatoscopy with CD31 and D2-40 expression revealed specific patterns and histological changes, aiding in the disease’s diagnosis (26).

The hypothesis of this study centered on exploring the potential correlations between dermatoscopic features and the expression of specific immunohistochemical markers (CD34, CD31, Melan A, and D2-40) in Basal Cell Carcinoma (BCC).

The researchers hypothesized that these correlations could provide insights into the angiogenic potential, melanocytic activity, and lymphangiogenesis within BCC lesions, thereby enhancing the understanding of BCC’s biological behavior and potentially influencing diagnostic and therapeutic strategies. Despite the well-formulated hypothesis and the comprehensive approach undertaken, the study predominantly found a lack of significant correlations between the dermatoscopic features and the expressions of the targeted immunohistochemical markers. The only notable exception of a correlation between dermoscopy observed is hemorrhage and the Melan-A marker.

The morphological characteristics of vascular formations in basal cell carcinomas appear to be primarily influenced by the histological location of the tumor and its dissemination pattern (27–29). The quantity of these vessels may correlate with the expression levels of CD31 and CD34 proteins (25). However, based on our observations, the morphological character of these vessels, specifically their shape as observed in flat, horizontal dermoscopic images, does not correlate with the expression of these proteins. This is because protein expression is related to their quantitative presence, indicating that both new and old vessels can exhibit any geometric shape. Therefore, vessels may present as branching linear, looped linear, curved linear, dot-like, and virtually any other type of vessel can correlate with these antigens. As for podoplanin, which is associated with the expression of the lymphatic system, lymphatic vessels in such superficial tumors are often not visible dermatoscopically, and there is usually difficulty in applying dermatoscopic diagnosis to these features (26, 30). This may indeed explain why there is no correlation in this aspect. Regarding Melan A, indicative of the presence of melanin, it was hypothesized that it would correlate with pigmented forms of carcinomas, where small and large gray-blue clumps and fine granularities at the periphery are observed. However, it has been determined that the amount of melanin in the samples is not significant. This could be since histopathological evaluations are generally based on single sections that are four micrometers thick and do not comprehensively traverse through the specific dermoscopic structures. In future research, a more detailed examination regarding the presence of melanin in different tumor areas might reveal a correlation. Nonetheless, it is essential to remember that the outcome of histopathological studies is usually based on a single slide made from a single cut, typically through the center, which may not encompass the relevant dermoscopic and histological structures. Furthermore, the Melan-A marker is indicative of melanosomal maturation. The presence of hemorrhage and its correlation with Melan-A might suggest an incidental or secondary association rather than a direct causative relationship. The observed significance could be due to the complex interplay of factors within the tumor microenvironment, which may not be fully captured by single-marker analysis.

The observation of a limited correlation between dermatoscopic features and the expression levels of CD31, CD34, Melan-A, and podoplanin in BCC could suggest that the angiogenic, melanocytic, and lymphangiogenic activities within BCC lesions are more complex than initially anticipated. This complexity might be influenced by the tumor’s microenvironment, which includes factors not directly related to these markers but could significantly impact their expression and the tumor’s vascular and lymphatic networks. Future studies might benefit from incorporating additional markers that could be implicated in these pathways or using more advanced imaging techniques to explore this complexity further. The exploratory nature of this study sets the stage for a deeper investigation into the biological behavior of BCC. While the lack of significant correlations between dermatoscopic features and the expressions of targeted immunohistochemical markers presents challenges, it also opens new avenues for research. Understanding the intricate interactions within the tumor microenvironment and identifying new biomarkers or imaging modalities could enhance diagnostic accuracy and therapeutic approaches for BCC in the future.

## Conclusions

Our study underscores the intricate relationship between dermatoscopic features and immunohistochemical markers in Basal Cell Carcinoma (BCC), revealing notably limited correlations between the two. The morphological characteristics of vascular formations in BCC are influenced more by the tumor’s location and dissemination pattern than by the expression levels of angiogenic markers CD31 and CD34. The difficulty in visualizing dermoscopy features linked with the lymphatic system and the absence of significant melanin presence in histopathological samples underscore the limitations of current dermatoscopic and histopathological evaluation techniques. This research suggests a need for advanced imaging and more comprehensive histopathological examination methods to accurately assess the underlying biology of BCC.

## Data Availability

The raw data supporting the conclusions of this article will be made available by the authors, without undue reservation.
